# Influencing consumer perceptions in green tourism: criteria and strategies for effective destination branding

**DOI:** 10.1371/journal.pone.0319254

**Published:** 2025-02-24

**Authors:** Tianchang Chen, Gao Liu, Xin Sui, Yasir Ahmed Solangi

**Affiliations:** 1 School of Humanities and Social Science, Yancheng Institute of Technology, Yancheng Jiangsu, China; 2 Renewable Energy Lab, College of Engineering, Prince Sultan University, Riyadh, Saudi Arabia; University of Economics Ho Chi Minh City, VIET NAM

## Abstract

As the global shift towards sustainable tourism intensifies, the need for effective destination branding strategies becomes increasingly important. This study investigates and prioritizes these strategies to enhance consumer perceptions of sustainable tourism destinations, with a focus on Guilin. The research examines five key criteria, twenty sub-criteria, and nine strategic approaches essential for destination branding. The primary objective is to shape consumer perceptions and promote green tourism marketing in the region. Through the application of Fuzzy Analytical Hierarchy Process (AHP) and Fuzzy Technique for Order Preference by Similarity to Ideal Solution (TOPSIS), key criteria and strategies are identified and evaluated. Results from the fuzzy AHP highlight communication and brand transparency (GM3) as the most critical criterion, stressing the necessity of transparent communication channels. Environmental sustainability (GM1) ranks as the second most important criterion, emphasizing the importance of eco-friendly practices and the preservation of Guilin’s natural environment. Stakeholder collaboration and partnerships (GM4) is identified as the third key criterion. The fuzzy TOPSIS analysis reveals the most effective strategies, with strategic collaborations and partnerships (STM9), eco-friendly infrastructure development (STM1), and energy efficiency measures (STM4) emerging as the top approaches. These strategies have significant potential to influence consumer perceptions positively towards green tourism marketing.

## 1. Introduction

In an era of heightened global environmental awareness, the tourism industry faces a crucial moment in balancing economic growth with environmental and social responsibility [1]. We are witnessing the rise of green tourism, a paradigm shifts where destinations blend economic prosperity with sustainable practices, catering to an expanding group of conscientious travelers [2]. Amidst this transformative landscape, the role of destination branding emerges as a linchpin, wielding the power to shape perceptions, attract tourists, and foster sustainable practices [3]. The urgency to align economic gains from tourism with environmental preservation is more pressing than ever. Destinations worldwide grapple with the imperative to not only cater to the diverse needs of tourists but also to mitigate the negative ecological consequences that often accompany mass tourism [4]. Global indicators and trends stress the imperative for sustainable practices. Facts from the United Nations world tourism organization (UNWTO) reveal an upward trajectory in international tourist arrivals, highlighting the industry’s enduring significance. However, alongside this growth, there is a growing recognition of the environmental toll that tourism can exact on host destinations [5]. Balancing these demands requires innovative approaches that not only generate immediate economic benefits but also safeguard long-term environmental sustainability, prompting the need for strategic frameworks like green tourism branding.

The World Tourism Organization’s (WTO) tourism highlights report succinctly summarizes this dual narrative, emphasizing the need for a paradigm shift toward sustainable tourism practices [6]. Eco-friendly tourism boosts both sustainability and economic growth by attracting sustainability-focused tourists, creating a positive cycle for the local economy. International reports consistently emphasize the multifaceted impact of sustainable tourism on various dimensions, ranging from economic values to environmental knowledge and public health [7]. As destinations embrace eco-friendly practices, there is a notable enhancement in their economic values, evident in increased GDP contributions. Moreover, the Global Sustainable Tourism Council emphasizes the core link between sustainable tourism and public health, stressing the significance of clean and well-maintained environments in nurturing the well-being of both local residents and visitors [8]. Clean environments promote healthier communities, making destinations more appealing, while destination branding helps position these places as sustainable leaders. In the pursuit of effective destination branding, these values collectively contribute to the creation of vibrant, resilient, and socially responsible tourism destinations. Furthermore, sustainable tourism initiatives help preserve cultural heritage and empower local communities, generating lasting socio-economic advantages. This approach ensures that destinations not only thrive economically but also maintain their ecological integrity, securing a sustainable future for both residents and visitors.

This study aims to assess the criteria and strategies inherent in destination branding for green tourism marketing in Guilin, China. Guilin, renowned for its unique karst landscapes and rich cultural heritage, offers a compelling case study for exploring green tourism branding strategies in a region with high environmental and touristic value. Therefore, this research utilizes the fuzzy Analytical Hierarchy Process (AHP) and fuzzy Technique for Order Preference by Similarity to Ideal Solution (TOPSIS) methods to assess and rank the criteria, sub-criteria, and strategies for destination branding to influence consumer perception for green tourism marketing. Fuzzy AHP is employed to decompose the complex decision-making problem into a hierarchical structure, enabling a systematic comparison of criteria and sub-criteria by incorporating expert judgments. This method addresses the inherent uncertainty and subjectivity in human decision-making [9]. On the other hand, fuzzy TOPSIS is used to rank the alternative strategies by measuring their relative closeness to the ideal solution. This method is particularly effective in handling the uncertainty and ambiguity of real-world data, allowing for a more accurate assessment of which strategies are best aligned with green tourism goals. These decision-making approaches promise a comprehensive understanding of the criteria and strategies involved in destination branding for green tourism marketing. This research not only addresses the factors and opportunities in green tourism marketing but also provides a model that can be adapted to other destinations seeking to balance tourism growth with environmental sustainability.

## 2. Literature Review

A foundational study by Kumar and Christodoulopoulou [10] examines the complex relationship between destination branding and sustainability, positing that a destination’s brand image significantly influences tourists’ decisions, especially when sustainability is a key consideration. This aligns with the principles of the Triple Bottom Line framework Zaharia and Zaharia [11], which advocates for the harmonious integration of economic, environmental, and social aspects in decision-making. Building on this foundation, Banerjee [12] emphasize the significance of destination branding in shaping tourist perceptions of sustainability, pointing out that a destination’s green initiatives directly impact its appeal to eco-conscious travelers. Moreover, the recent studies highlight the growing significance of green marketing strategies in enhancing tourist satisfaction and loyalty in Chinese eco-destinations [13,14]. A study by Mengkebayaer et al. [15] focuses on eco-destination loyalty, emphasizing the roles of perceived value and tourist experience in forming destination attachment and equity. The research shows that these factors significantly contribute to destination loyalty, indicating that effective destination branding can lead to increased tourist retention and satisfaction. Another study by Jeong and Kim [16] explores the structural relationships between event quality, destination image, perceived value, tourist satisfaction, and destination loyalty. Their findings highlight the powerful impact of perceived value and destination image on tourist satisfaction, which in turn enhances destination loyalty. The study by Vilkaite-Vaitone [17] investigates the impact of social media influencers on sustainable consumption, highlighting how their credibility and perceived importance positively affect consumers’ sustainable behaviors. Another study focuses on the role of social media in sustainable tourism within Indian states, particularly Assam and Odisha [18]. It highlights the effectiveness of social media campaigns in spreading awareness and promoting sustainable tourist destinations.

### 2.1. Destination branding and perception

Destination branding plays a pivotal role in shaping consumer perceptions and influencing travel decisions [19,20]. In recent years, there has been a growing recognition of the importance of destination branding, particularly in the context of green tourism marketing [21]. One of the key aspects of destination branding is the perception of authenticity. Travelers are increasingly seeking authentic experiences that allow them to connect with the local culture, heritage, and environment. Destination branding efforts that authentically reflect the unique characteristics of a destination, such as its natural landscapes, cultural traditions, and community values, are more likely to resonate with travelers and build a positive perception of the destination [22]. In addition to authenticity, destination branding also influences perceptions of quality and value. Travelers associate destination brands with certain attributes, such as luxury, adventure, or relaxation, based on their previous experiences. Perceptions of destination brands are shaped by various factors, including marketing communications, word-of-mouth recommendations, and personal experiences. Positive interactions with a destination, such as memorable travel experiences or interactions with friendly locals, can enhance perceptions of the destination brand and lead to repeat visits and positive word-of-mouth. Conversely, negative experiences or perceptions, such as environmental degradation, overcrowding, or cultural insensitivity, can damage a destination’s brand reputation and deter travelers from visiting [23]. Therefore, destination branding efforts must align with sustainable tourism principles and prioritize responsible tourism practices to ensure the long-term viability of the destination and its brand image.

### 2.2. Emerging trends in destination branding

The traditional paradigms of destination branding are being redefined, with scholars increasingly recognizing the multifaceted impact that sustainable practices wield on shaping tourists’ perceptions. A recent study by Chen et al. [24] investigates the emerging trends, highlighting the growing importance of authenticity in destination branding narratives. Authenticity, they argue, has become a critical factor influencing how consumers perceive a destination’s commitment to green tourism, emphasizing the need for transparent and genuine communication in branding strategies. This trend aligns with the broader global movement towards responsible and ethical tourism, as outlined in a meta-analysis by Bianchi and Gonzalez [25], which emphasizes the role of authenticity in creating meaningful connections between destinations and eco-conscious consumers in Chile. Moreover, the integration of technology in destination branding has become a focal point of exploration. Studies by Gulati [26] and Seyfi et al. [27] plunge into the influence of digital platforms and social media on shaping consumer perceptions of destination sustainability. Emerging technologies not only function as communication channels but also facilitate real-time interactions, empowering destinations to spotlight their green initiatives and directly engage with eco-conscious travelers. This technological transition is recognized as a key driver in the destination branding, providing new opportunities to establish and convey their dedication to green tourism.

A noteworthy development in the literature also revolves around the concept of co-creation in destination branding. Scholars like Tøttenborg et al. [28] argue that involving tourists in the destination branding process fosters a sense of ownership and shared responsibility for sustainability. This co-creation trend marks a departure from traditional top-down approaches, highlighting the potential for collaborative efforts between destinations and consumers to shape and enhance the perception of green tourism destinations. Within the Chinese context, the literature on green tourism and destination branding has witnessed a surge in interest. Zheng et al. [29] scrutinize the sustainable development of Chinese tourism destinations, emphasizing the need for effective destination branding strategies to align with green principles. Furthermore, Qingyun and Sarkis [30] investigate into the role of green marketing in enhancing the competitiveness of Chinese tourist destinations, shedding light on how eco-friendly initiatives can be leveraged within destination branding efforts. These insights align with the broader global trends, as outlined in a comprehensive meta-analysis by Wu et al. [31], which stresses the increasing importance of sustainable practices in tourism, positioning destination branding as a pivotal factor in navigating this shift.

### 2.3. Case of Guilin

**Fig 1:**
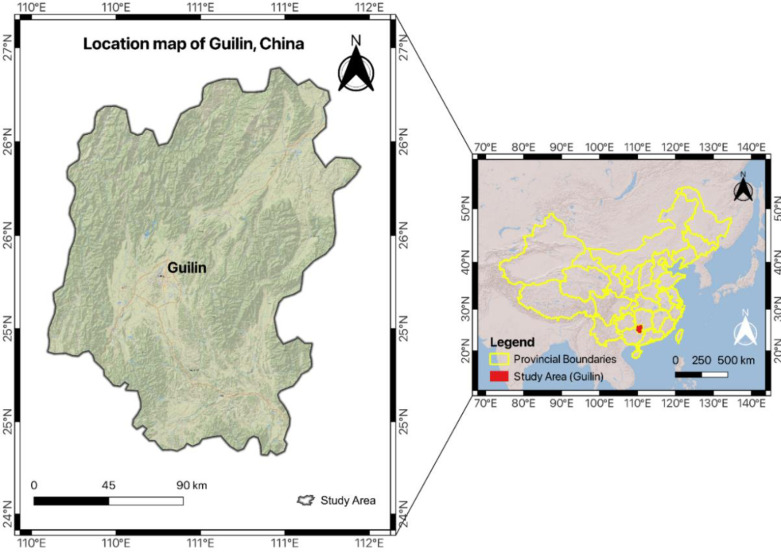
The geographical map of Guilin, China [Source: authors own elaboration].

The choice of Guilin, China as the focal point of this study is strategic, given its renowned status for captivating landscapes and cultural richness within the Chinese tourism landscape. Guilin, located in the southern region of China, Fig 1 presents the geographical map of the region. The country’s unwavering commitment to green tourism, as emphasized by reports from the China National Tourism Administration, aligns strategically with the principles of green marketing, recognizing the profound impact of sustainability on consumer preferences in the contemporary landscape [32]. Within this dynamic context, Guilin emerges as a compelling case study for destination branding, green tourism, and consumer perception within the broader context of China. The focus of this study is to navigate the complexities of these dynamics, with a particular focus on Guilin, explaining the criteria and strategies inherent in destination branding for green tourism marketing in the Chinese landscape. Despite the valuable contributions of prior studies [33–36], there remains a research gap in the specific dimensions of destination branding for green tourism marketing in certain regions, such as Guilin. Existing literature has mainly focused on Guilin’s commitment to sustainability without examining how branding strategies align with and enhance consumer perceptions in the context of green tourism.

Guilin’s initiatives towards sustainable tourism, providing insights into the destination’s efforts to balance economic growth with environmental preservation [37]. This aligns with the study by Chen et al. [20], who analyze the role of destination branding in sustainable tourism development, emphasizing the need for destinations like Sanya to craft authentic narratives that resonate with eco-conscious travelers. However, there remains a research gap concerning the specific criteria and strategies employed in destination branding for green tourism marketing within Guilin, which this study aims to address using the fuzzy AHP and fuzzy TOPSIS methodologies. The fuzzy AHP method is used to evaluate the key criteria and sub-criteria, while the fuzzy TOPSIS method is used to rank the strategies. Thus, in the study, the comprehensive literature review has been conducted to identify the key criteria and strategies, which are crucial for the development for the destination branding and its influence on consumer perception for green tourism marketing.

### 2.4. Identification of criteria and sub-criteria

The identification of criteria and sub-criteria plays a vital role in shaping consumer perceptions and fostering sustainable practices. Therefore, in this study, the thorough literature review has been examined to identify and determine the most crucial criteria. The selection of these identified criteria and sub-criteria is listed in Table 1.

**Table 1:** Identified criteria and sub-criteria.

Criteria	Sub-criteria	Description	Reference
Environmental sustainability (GM1)	Green infrastructure (GM11)	Emphasizes the integration of sustainable and eco-friendly infrastructure to minimize environmental impact.	[38]
	Biodiversity conservation (GM12)	Focuses on preserving and protecting the rich biodiversity of the destination as a key element of sustainability.	[39]
	Waste management (GM13)	Involves effective waste management practices to reduce environmental pollution and promote responsible tourism.	[40]
	Energy efficiency (GM14)	Prioritizes the adoption of energy-efficient measures to minimize the ecological footprint and promote sustainable energy use.	[41]
Community engagement (GM2)	Local community involvement (GM21)	Advocates for active participation and collaboration with the local community in tourism initiatives.	[42]
	Cultural preservation (GM22)	Stresses the importance of preserving and safeguarding the cultural heritage of the destination.	[43]
	Social impact initiatives (GM23)	Focuses on implementing initiatives that contribute positively to the social well-being of the local community.	[26]
	Inclusive tourism practices (GM24)	Promotes inclusivity in tourism, ensuring that all segments of the community benefit from tourism activities.	[44]
Communication and brand transparency (GM3)	Information transparency (GM31)	Promotes open and transparent communication of information regarding sustainable practices and policies.	[45]
	Open communication channels (GM32)	Advocates for accessible communication channels between tourists, stakeholders, and the destination management.	[46]
	Responsiveness to visitor feedback (GM33)	Emphasizes the importance of actively responding to and incorporating feedback from visitors.	[47]
	Clarity in sustainability messaging (GM34)	Stresses the need for clear and coherent communication of the destination’s sustainability messaging to enhance understanding.	[48]
Stakeholder collaboration and partnerships (GM4)	Collaboration with local businesses (GM41)	Advocates for partnerships and collaboration with local businesses to enhance the overall tourism experience.	[49]
	Partnerships with environmental agencies (GM42)	Encourages alliances with environmental organizations to strengthen sustainability initiatives.	[50]
	Engagement with government agencies (GM43)	Stresses the importance of engaging with government agencies to align tourism strategies with broader sustainable development goals.	[51]
	Collaboration with international sustainability initiatives (GM44)	Promotes collaboration with international initiatives to gain insights and support for global sustainability standards.	[52]
Authenticity and cultural relevance (GM5)	Authentic cultural experiences (GM51)	Encourages the offering of genuine and immersive cultural experiences to visitors.	[53]
	Preservation of heritage (GM52)	Highlights the importance of preserving and maintaining the cultural and historical heritage of the destination.	[54]
	Representation of local traditions (GM53)	Stresses the need to authentically represent and celebrate local traditions in tourism offerings.	[55]
	Integration of indigenous practices (GM54)	Encourages the integration of indigenous practices into tourism experiences to enhance cultural authenticity.	[56]

### 2.5. Identification of strategies for sustainable destination branding

In this research, the comprehensive literature review has been examined in order to identify and assess the critical strategies aimed at propelling Guilin into a sustainability within destination branding. Therefore, we identified nine key strategies for sustainable destination branding to influence consumption perception in Guilin. Table 2 displays nine identified strategies for sustainable destination branding in Guilin.

**Table 2:** Proposed strategies.

Strategies	Overview	Reference
Eco-friendly infrastructure development (STM1)	Invest in and promote the development of green infrastructure, such as sustainable buildings, energy-efficient transportation, and eco-friendly amenities, to enhance the overall environmental sustainability of Guilin.	[57]
Biodiversity conservation initiatives (STM2)	Implement programs and policies aimed at preserving and protecting the rich biodiversity of Guilin, including conservation projects, wildlife protection measures, and sustainable land use planning.	[58]
Waste reduction and recycling programs (STM3)	Introduce comprehensive waste management initiatives, including recycling programs, waste reduction campaigns, and sustainable disposal methods, to minimize the environmental impact of tourism activities.	[59]
Energy efficiency measures (STM4)	Implement energy-efficient practices across tourism facilities, accommodations, and transportation services in Guilin, utilizing renewable energy sources and adopting energy-saving technologies.	[41]
Community empowerment and involvement (STM5)	Foster active involvement of the local community in tourism decision-making processes, ensuring their participation in sustainable tourism initiatives, and promoting cultural exchange between tourists and locals.	[60]
Cultural heritage preservation programs (STM6)	Develop and implement initiatives focused on preserving cultural heritage, including historical sites, traditional practices, and cultural events, to enhance authenticity and cultural relevance.	[61]
Social impact and inclusivity campaigns (STM7)	Launch campaigns and initiatives that have a positive social impact, such as community development projects, support for local businesses, and inclusive tourism practices that cater to diverse visitor segments.	[62]
Transparent communication and education (STM8)	Establish clear and transparent communication channels to convey sustainability efforts and educate tourists about commitment to green tourism. This includes informative signage, online platforms, and educational programs.	[63]
Strategic collaborations and partnerships (STM9)	Form collaborations and partnerships with local businesses, environmental agencies, government entities, and international sustainability initiatives to strengthen position as a leader in green tourism and sustainable destination management.	[64]

## 3. Methodology

**Fig 2:**
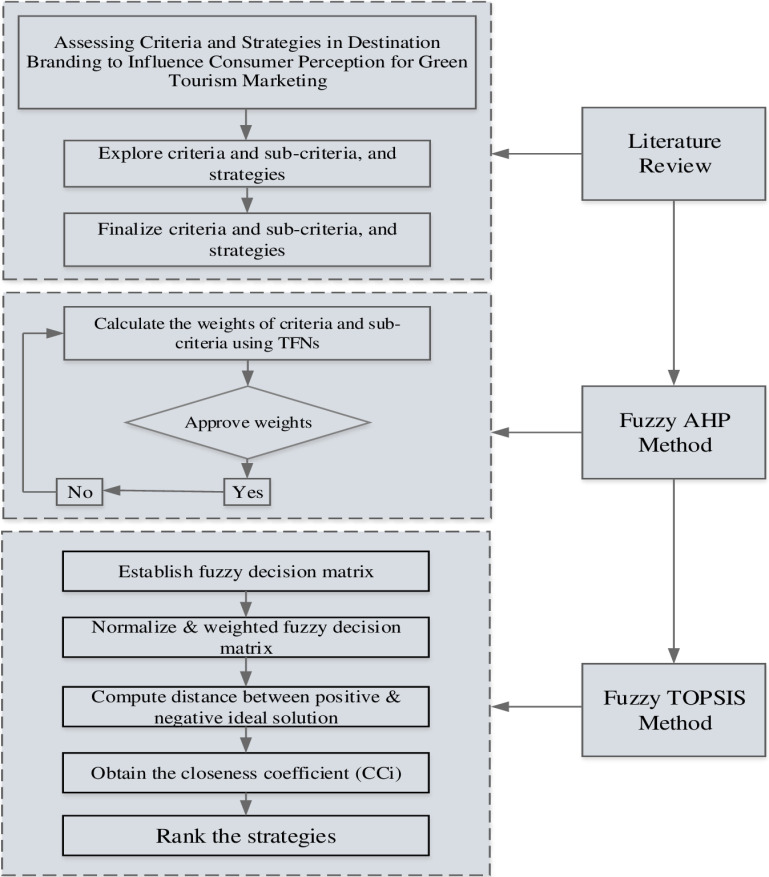
Research methodology.

The research design for the study is outlining the systematic approach employed to investigate destination branding strategies for green tourism marketing in Guilin. Initially, the comprehensively literature review was conducted to identify the key criteria, sub-criteria, and strategies. Secondly, the fuzzy AHP method has been used to evaluate the criteria and sub-criteria that influence the consumer perception in green tourism market in Guilin, China. Next, the fuzzy TOPSIS method has been used to rank the strategies for adoption of brand destination in green tourism market in Guilin. Fig 2 displays the research framework of this study.

### 3.1. Fuzzy AHP

The AHP was developed by Saaty in 1970s [65]. This method retains hierarchical structure, systematically organizing criteria and sub-criteria for evaluation. In the study, the fuzzy AHP method is used, which effectively handles subjective criteria and preferences in decision-making, addressing imprecise or vague information [66]. This research used the pairwise comparison matrix employed using triangular fuzzy numbers (TFNs). This scale used in this research is presented in Table 3.

**Table 3:** TFNs scale.

No.	Linguistic Variables	TFNs
1	Just equal	(1,1,11)
2	Equally important	(0.5,1,1.5)
3	Weakly important	(1,1.5,2)
4	Strongly important	(1.5,2,2.5)
5	Very strongly important	(2,2.5,3)
6	Extremely preferred	(2.5,3,3.5)

In this research, a series of steps were undertaken to determine the inconsistency ratio of the fuzzy pairwise comparison matrix [67].

Step I. Transform a triangular fuzzy matrix into two independent matrices:


$$X_{i}=\left(l_{i},m_{i},u_{i}\right)$$ (1)

Next, using the middle fuzzy triangular matrix, the first triangular fuzzy matrix can be created:


$$X_{m}=\left[x_{ijm}\right]$$ (2)

Here, the second triangular fuzzy matrix can be created using the geometric mean approach for the upper and lower limits of the TFNs:


$$X_{g}=[\sqrt{x_{iju}x_{ijl}}$$ (3)

Step II. Construct the weight vector based on the Saaty approach and computation of lambda max.

Step III. Create consistency index (CI):


$$CI_{m}=\frac{\lambda_{max}^{m}-n}{n-1}$$ (4)

$$CI_{g}=\frac{\lambda_{max}^{g}-n}{n-1}$$ (5)

Step IV. Create a consistency ratio (CR) for every matrix. The random index (RI) of each matrix is divided by its consistency index (CI) for CR:


$$CR_{m}=\frac{CI_{m}}{RI_{m}}$$ (6)

$$CR_{g}=\frac{CI_{g}}{RI_{g}}$$ (7)

### 3.2. Fuzzy TOPSIS

The TOPSIS method is used to identify the most favorable and least favorable gaps between ideal and undesirable solutions, as emphasized by Shih et al. [68]. This approach proves valuable in addressing complex and uncertain issues, prompting the adoption of the fuzzy TOPSIS method in this study. The following are the key steps of fuzzy TOPSIS method [69]:

Step I. Define the fuzzy decision matrix *X*.


$$X={\left(x_{ij}\right)}_{m\times n}$$ (8)

Step II. Establish the normalized fuzzy decision matrix (for benefit and cost criteria).


$$R={\left[r_{ij}\right]}_{m\times n}$$ (9)

$$r_{ij}=\left(\frac{a_{ij}}{c_{j}^{+}},\frac{b_{ij}}{c_{j}^{+}},\frac{c_{ij}}{c_{j}^{+}}\right)$$ (10)

Where, $c_{j}^{+}=\max c_{ij}$ (Benefit criteria)


$$r_{ij}=\left(\frac{a_{j}^{-}}{c_{ij}},\frac{a_{j}^{-}}{b_{ij}},\frac{a_{j}^{-}}{a_{ij}}\right)$$ (11)

$a_{j}^{-}=mina_{ij}$ (Cost criteria)

Step III. Calculate the weighted normalized fuzzy decision matrix.


$$V={\left[v_{ij}\right]}_{m\times n}$$ (12)

Here, $v_{ij}=r_{ij}\times w_{j}$

Step IV. Compute the distance of each alternative from the fuzzy $d^{+}$ and fuzzy $d^{-}$ ideal solution:


$$d_{i}^{+}=\overset{n}{\underset{j=1}{\sum }}d\left(v_{ij}-v_{j}^{+}\right)$$ (13)

Where, j=1,2,3,…,m

And fuzzy negative solution:


$$d_{i}^{-}=\overset{n}{\underset{j=1}{\sum }}d\left(v_{ij}-v_{j}^{-}\right)$$ (14)

Where, j=1,2,3,…,m

Step V. Compute the Closeness Coefficient $\left(CC_{i}\right)$.


$$CC_{i}=\frac{d_{i}^{-}}{d_{i}^{+}+d_{i}^{-}}$$ (15)

Step VI. Select the alternative with the highest $CC_{i}$ score.

Guilin - known for its unique nature and sustainable development has been taken as an example that illustrates how the determined indicators and directions are expressed in reality. Through the condition of selecting Guilin which is famed for its endeavors driven for environmental sustainability, the case study attempts to extract the implication of the tangled network of the criteria and the strategies that make Guilin an identifying tourism haven observed worldwide as an attraction for sustainable tourism. In this study, the six experts were consulted via email to provide their opinion on the questionnaire survey. The experts primarily from China, in the field of academia, government, and policymakers.

## 4. Results and analysis

**Fig 3:**
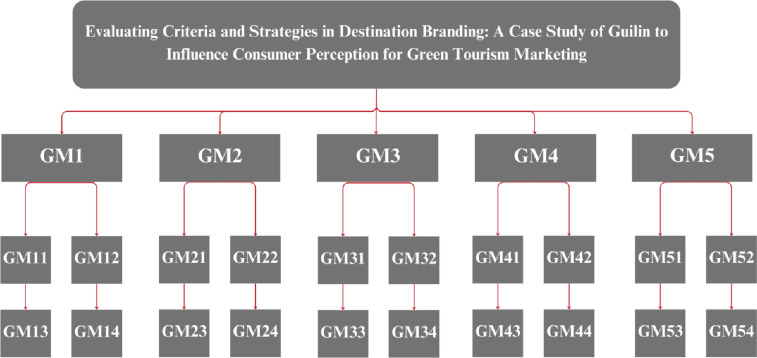
Hierarchical representation.

This section unveils the findings of thorough research on destination branding for green tourism marketing in Guilin, China. It examines the results derived from the Fuzzy AHP and Fuzzy TOPSIS methodologies, focusing on five primary criteria and twenty sub-criteria, complemented by nine strategies, to explore sustainable tourism practices. The hierarchical representation of the research is depicted in Fig 3.

### 4.1. Results of Fuzzy AHP (main-criteria)

**Fig 4:**
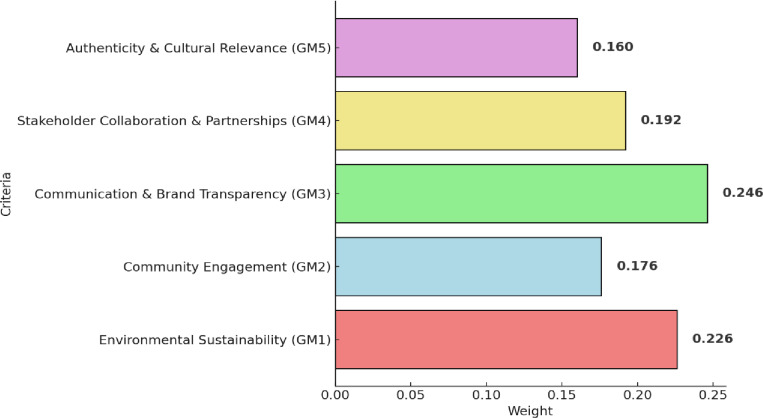
Results of main criteria.

Table 4 presents the integrated fuzzy pairwise comparison matrix of five criteria. While the results of these criteria is given in Fig 4. The findings indicate that communication and brand transparency (GM3) emerge as the most critical, obtaining the highest weight. This emphasizes the paramount importance of transparent communication regarding sustainability efforts, aligning with global best practices. Existing report, such as those by UNWTO [5] on sustainable tourism communication, support the significance of effective communication in sustainable tourism initiatives. Following closely is environmental sustainability (GM1), highlighting the significance of eco-friendly practices and the preservation of Guilin’s natural environment. This resonates with Mastika and Nimran [70] emphasis on ecological considerations in destination branding. Stakeholder collaboration and partnerships (GM4) secure the third position, accenting collaborative efforts involving local businesses, environmental agencies, and international sustainability initiatives, in alignment with studies promoting multi-stakeholder engagement in sustainable tourism. community engagement (GM2) secures the fourth spot, indicating the importance of involving the local community in sustainable tourism initiatives. Yrza and Filimonau [71] study on community engagement in sustainable tourism provides insights into the positive impact of community involvement. Lastly, authenticity and cultural relevance (GM5) holds the fifth position, stressing the significance of preserving Guilin’s cultural heritage.

**Table 4:** The fuzzy pairwise comparison matrix main criteria.

	GM1	GM2	GM3	GM4	GM5
**GM1**	(1, 1, 11)	(1.156, 1.284, 1.413)	(0.827, 0.919, 1.011)	(1.059, 1.177, 1.295)	(1.271, 1.412, 1.554)
**GM2**	(0.701, 0.779, 0.857)	(1, 1, 11)	(0.644, 0.715, 0.787)	(0.825, 0.917, 1.008)	(0.99, 1.1, 1.21)
**GM3**	(0.979, 1.088, 1.197)	(1.258, 1.398, 1.538)	(1, 1, 11)	(1.153, 1.281, 1.409)	(1.383, 1.537, 1.691)
**GM4**	(0.765, 0.85, 0.935)	(0.982, 1.091, 1.2)	(0.702, 0.78, 0.859)	(1, 1, 11)	(1.08, 1.2, 1.32)
**GM5**	(0.637, 0.708, 0.779)	(0.818, 0.909, 1)	(0.585, 0.65, 0.715)	(0.75, 0.833, 0.917)	(1, 1, 11)

### 4.2. Results of Fuzzy AHP (sub-criteria)

**Fig 5:**
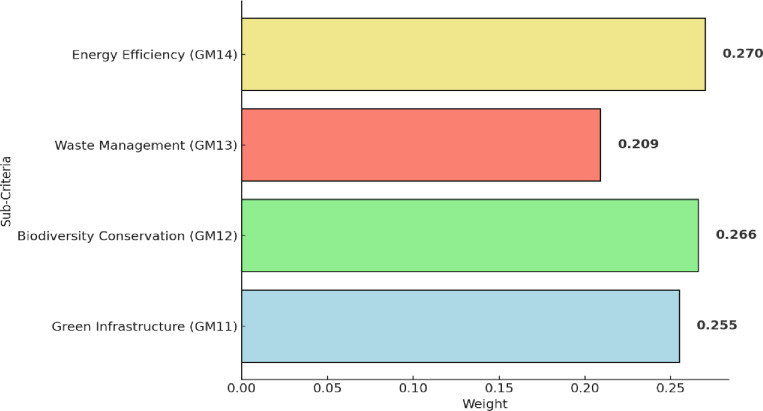
Results of sub-criteria within environmental sustainability (GM1).

Table 5 shows the integrated fuzzy pairwise comparison matrix of sub-criteria of environmental sustainability (GM1). The ranking of these sub-criteria is presented in Fig 5. Within this framework, energy efficiency (GM14) emerges with the highest ranked, emphasizing the city’s dedication to optimizing energy consumption. Notably, existing studies, such as those by Lu et al. [41], highlight the vital role of effective energy management in enhancing the sustainability of tourist destinations, aligning with Guilin’s prioritization. Biodiversity conservation (GM12) secures the second-ranked, reflecting Guilin’s commitment to preserving its rich biodiversity. Studies by Schloegel [72] further support this focus on biodiversity conservation, highlighting its significance in ecotourism and sustainable destination practices. Green infrastructure (GM11) follows closely, highlighting Guilin’s endeavor to develop sustainable built environments. Waste management (GM13), stresses the importance of effective waste management practices within Guilin’s sustainable tourism framework. Research by Tsai et al. [73] emphasizes the crucial role of waste management in minimizing environmental impact in tourist destinations.

**Table 5:** The fuzzy pairwise comparison matrix sub-criteria of environmental sustainability (GM1).

	GM11	GM12	GM13	GM14
**GM11**	(1, 1, 11)	(0.863, 0.959, 1.055)	(1.098, 1.220, 1.342)	(0.850, 0.944, 1.039)
**GM12**	(0.939, 1.043, 1.147)	(1, 1, 11)	(1.145, 1.273, 1.400)	(0.887, 0.985, 1.084)
**GM13**	(0.738, 0.820, 0.902)	(0.707, 0.786, 0.864)	(1, 1, 11)	(0.697, 0.774, 0.851)
**GM14**	(0.953, 1.059, 1.165)	(0.914, 1.015, 1.117)	(1.163, 1.292, 1.421)	(1, 1, 11)

**Fig 6:**
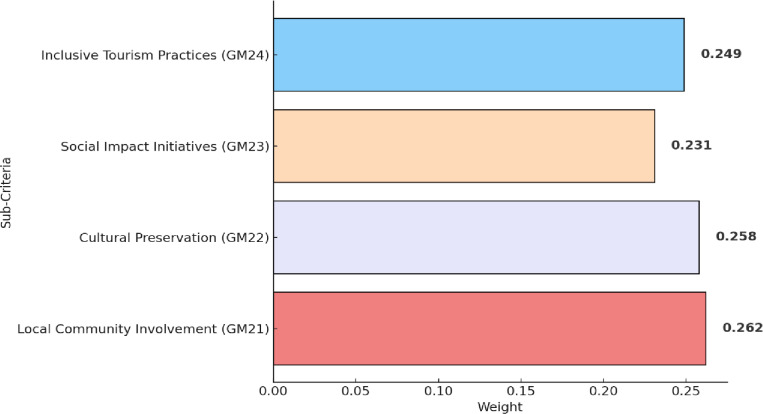
Results of sub-criteria within community engagement (GM2).

Table 6 depicts the fuzzy pairwise comparison matrix of sub-criteria associated with community engagement (GM2). The ranking of these sub-criteria is displayed in Fig 6. Local community involvement (GM21) claims the highest rank, featuring Guilin’s dedicated focus on actively engaging the local community in tourism decision-making processes. This aligns with existing studies, such as Sharma and Bhat [74], which emphasize the positive impacts of community involvement in sustainable tourism initiatives. Cultural preservation (GM22) secures the second-highest rank, highlighting Guilin’s commitment to preserving its cultural heritage. Study by Ghirardello et al. [75] support this emphasis on cultural preservation, emphasizing its integral role in sustainable destination branding. Inclusive tourism practices (GM24) follow closely, indicating Guilin’s dedication to ensuring tourism practices cater to diverse visitor segments, aligning with global trends advocating for inclusive tourism experiences. Social impact initiatives (GM23), reflect Guilin’s commitment to fostering positive social impacts through tourism. Research by Khalid et al. [76] underlines the importance of community-focused initiatives as integral components of sustainable tourism strategies.

**Table 6:** The fuzzy pairwise comparison matrix sub-criteria of community engagement (GM2).

	GM21	GM22	GM23	GM24
**GM21**	(1, 1, 11)	(0.914, 1.016, 1.117)	(1.021, 1.134, 1.248)	(0.947, 1.052, 1.157)
**GM22**	(0.886, 0.985, 1.083)	(1, 1, 11)	(1.005, 1.117, 1.229)	(0.933, 1.036, 1.140)
**GM23**	(0.794, 0.882, 0.970)	(0.806, 0.895, 0.985)	(1, 1, 11)	(0.835, 0.928, 1.020)
**GM24**	(0.855, 0.950, 1.045)	(0.869, 0.965, 1.062)	(0.970, 1.078, 1.186)	(1, 1, 11)

**Fig 7:**
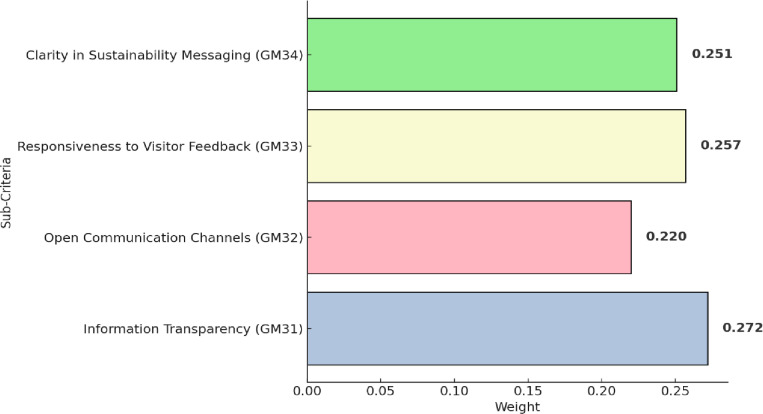
Results of sub-criteria within communication and brand transparency (GM3).

Table 7 demonstrates the fuzzy pairwise comparison matrix of sub-criteria related to communication and brand transparency (GM3). The prioritizing order of these sub-criteria is shown in Fig 7. Information transparency (GM31) achieved the first rank, highlighting the importance placed on providing clear and accessible information about sustainability efforts. This aligns with study by Tseng et al [77], emphasizing the crucial role of information transparency in sustainable tourism communication. Responsiveness to visitor feedback (GM33) secures the second topped place, highlighting Guilin’s dedication to actively addressing and incorporating visitor input into sustainability practices. Research by Stylidis et al. [78] support this emphasis on responsiveness, emphasizing its role in enhancing the adaptability and effectiveness of sustainable tourism initiatives. Clarity in sustainability messaging (GM34), indicating commitment to conveying sustainability messages in a clear and understandable manner. Open communication channels (GM32), reflect Guilin’s efforts to maintain accessible communication pathways.

**Table 7:** The fuzzy pairwise comparison matrix sub-criteria of communication and brand transparency (GM3).

	GM31	GM32	GM33	GM34
**GM31**	(1, 1, 11)	(1.113, 1.236, 1.360)	(0.953, 1.058, 1.164)	(0.975, 1.084, 1.192)
**GM32**	(0.728, 0.809, 0.890)	(1, 1, 11)	(0.770, 0.856, 0.942)	(0.789, 0.876, 0.964)
**GM33**	(0.850, 0.945, 1.039)	(1.051, 1.168, 1.285)	(1, 1, 11)	(0.922, 1.024, 1.126)
**GM34**	(0.831, 0.923, 1.015)	(1.027, 1.141, 1.255)	(0.879, 0.977, 1.074)	(1, 1, 11)

**Fig 8:**
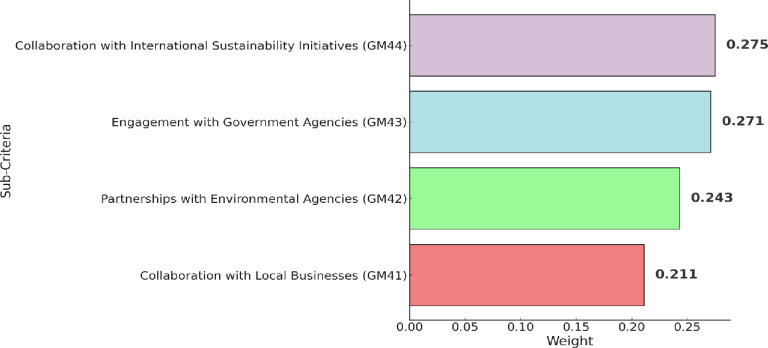
Results of sub-criteria within stakeholder collaboration and partnerships (GM4).

Table 8 presents the fuzzy pairwise comparison matrix of sub-criteria associated with stakeholder collaboration and partnerships (GM4). While the ranking of these sub-criteria is given in Fig 8. Collaboration with international sustainability initiatives (GM44) attained the first rank, emphasizing Guilin’s commitment to global sustainability efforts. This aligns with studies by Wall [79], highlighting the key role of international collaboration in advancing sustainability initiatives. Engagement with government agencies (GM43) secures the second-rank, showcasing Guilin’s dedication to collaborating with governmental entities for sustainable tourism practices. Study by Purnomo et al. [80] support this focus on governmental engagement, recognizing it as a key element in effective destination management. Partnerships with environmental agencies (GM42) appeared as second key sub-criteria, indicating Guilin’s emphasis on collaborating with environmental organizations. Finally, collaboration with local businesses (GM41), reflects Guilin’s recognition of the importance of local business involvement in sustainable tourism.

**Table 8:** The fuzzy pairwise comparison matrix sub-criteria of stakeholder collaboration and partnerships (GM4).

	GM41	GM42	GM43	GM44
**GM41**	(1, 1, 11)	(0.781, 0.868, 0.955)	(0.701, 0.779, 0.857)	(0.691, 0.767, 0.844)
**GM42**	(1.036, 1.152, 1.267)	(1, 1, 11)	(0.807, 0.897, 0.986)	(0.795, 0.884, 0.972)
**GM43**	(1.156, 1.284, 1.413)	(1.004, 1.115, 1.227)	(1, 1, 11)	(0.887, 0.985, 1.084)
**GM44**	(1.173, 1.303, 1.434)	(1.019, 1.132, 1.245)	(0.913, 1.015, 1.116)	(1, 1, 11)

**Fig 9:**
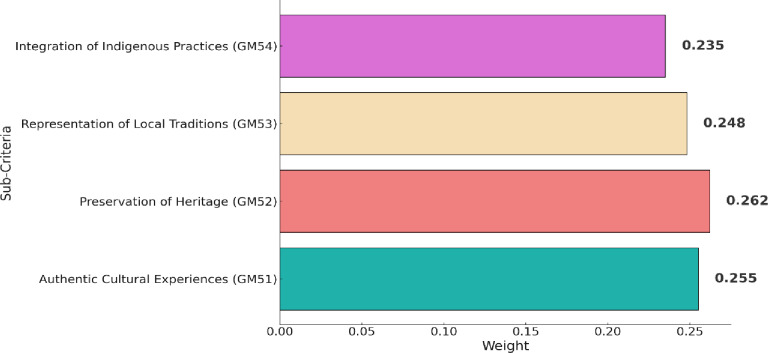
Results of sub-criteria within authenticity and cultural relevance (GM5).

Table 9 shows the fuzzy pairwise comparison matrix of sub-criteria associated with authenticity and cultural relevance (GM5). While the ranking of these sub-criteria is specified in Fig 9. Preservation of heritage (GM52) shows the highest weight, stressing Guilin’s dedicated commitment to preserving its rich cultural heritage. This aligns with existing studies, such as Zhao et al. [81], emphasizing the crucial role of heritage preservation in sustainable destination branding. Authentic cultural experiences (GM51) secure the second-ranked weight, highlighting Guilin’s emphasis on providing visitors with genuine cultural experiences. Research by Chambers [82] support this focus on authenticity, recognizing its significance in enhancing the overall cultural relevance of a destination. Representation of local traditions (GM53), indicating Guilin’s commitment to showcasing and celebrating local traditions. Lastly, integration of indigenous practices (GM54), reflects Guilin’s efforts to incorporate indigenous practices into its tourism offerings.

**Table 9:** The fuzzy pairwise comparison matrix sub-criteria of authenticity and cultural relevance (GM5).

	GM51)	GM52	GM53	GM54
**GM51**	(1, 1, 11)	(0.876, 0.973, 1.071)	(0.925, 1.028, 1.131)	(0.977, 1.085, 1.194)
**GM52**	(0.925, 1.027, 1.130)	(1, 1, 11)	(0.951, 1.056, 1.162)	(1.003, 1.115, 1.226)
**GM53**	(0.875, 0.973, 1.070)	(0.852, 0.947, 1.041)	(1, 1, 11)	(0.950, 1.055, 1.161)
**GM54**	(0.829, 0.922, 1.014)	(0.807, 0.897, 0.987)	(0.853, 0.948, 1.042)	(1, 1, 11)

### 4.3. Overall sub-criteria weight using fuzzy AHP

Based on the Fuzzy AHP results for the overall sub-criteria in Guilin’s destination branding strategy, several insights emerge regarding the prioritization of factors critical to sustainable tourism. Table 10 presents the ranking of overall sub-criteria. The finding show that information transparency (GM31) holds the highest importance, signifying the importance of providing clear, accessible information about sustainability efforts. This reflects global trends emphasizing the necessity of open communication in building trust and engagement with visitors. Similarly, responsiveness to visitor feedback (GM33) ranks second, highlighting the adaptive value of incorporating visitor input. Besides, clarity in sustainability messaging (GM34) ranks third, further emphasizing the essential role of clear communication. This suggests that Guilin’s focus on transparent and responsive engagement is pivotal in its sustainable branding. Energy efficiency (GM14) ranks fourth overall, indicating Guilin’s commitment to optimizing energy use, a key aspect of reducing the environmental footprint. Biodiversity conservation (GM12), ranked fifth, emphasizes efforts to protect Guilin’s unique ecosystems. Likewise, waste management (GM13) and partnerships with local businesses (GM41) rank towards the bottom, highlighting areas that may require increased emphasis if Guilin aims to address comprehensive sustainable waste practices and stronger local economic integration. Lastly, social impact initiatives (GM23) and inclusive tourism practices (GM24) are also relatively lower in the rankings, suggesting an area where Guilin might further develop its approach to social equity and diversity in tourism.

**Table 10:** The ranking and global weight of overall sub-criteria.

Sub-Criteria	Global Weight	Rank
Information Transparency GM31)	0.0669	1
Responsiveness to Visitor Feedback (GM33)	0.0632	2
Clarity in Sustainability Messaging (GM34)	0.0617	3
Energy Efficiency (GM14)	0.0610	4
Biodiversity Conservation (GM12)	0.0601	5
Green Infrastructure (GM11)	0.0576	6
Open Communication Channels (GM32)	0.0541	7
Collaboration with International Sustainability Initiatives (GM44)	0.0528	8
Engagement with Government Agencies (GM43)	0.0520	9
Waste Management (GM13)	0.0472	10
Partnerships with Environmental Agencies (GM42)	0.0467	11
Local Community Involvement (GM21)	0.0461	12
Cultural Preservation (GM22)	0.0454	13
Inclusive Tourism Practices (GM24)	0.0438	14
Preservation of Heritage (GM52)	0.0419	15
Authentic Cultural Experiences (GM51)	0.0408	16
Social Impact Initiatives (GM23)	0.0407	17
Collaboration with Local Businesses (GM41)	0.0405	18
Representation of Local Traditions (GM53)	0.0397	19
Integration of Indigenous Practices (GM54)	0.0376	20

### 4.4. Results of strategies using fuzzy TOPSIS

This study used the fuzzy TOPSIS method to assess and prioritize the nine identified strategies aimed at influencing consumer perception for green tourism marketing in Guilin. The selection of these strategies is guided by the primary goal of enhancing Guilin’s destination branding and fostering sustainable tourism practices. The fuzzy TOPSIS analysis ranks the strategies based on their performance in influencing consumer perception for green tourism marketing in Guilin. Table 11 shows the results of these strategies based on fuzzy TOPSIS. The results indicate that strategic collaborations and partnerships (STM9) is ranked first strategy due to its alignment with critical criteria such as stakeholder collaboration and partnerships, which are deemed essential for sustainable tourism development. Previous research, such as Kisi [83], has highlighted the importance of strategic partnerships in sustainable tourism development, aligning with our findings that emphasize the value of such collaborations. Following closely, eco-friendly infrastructure development (STM1) has been highlighted as critical, suggesting that investments in sustainable infrastructure are pivotal for enhancing Guilin’s environmental sustainability as a tourist destination. This is supported by studies like Kwag et al. [84], which also stress the importance of sustainable infrastructure investments. In our analysis, energy efficiency measures (STM4) and biodiversity conservation initiatives (STM2) are ranked third and fourth, respectively, indicating their significant contributions to sustainable tourism in Guilin. Thus, in the earlier studies authors emphasized the positive impacts of energy efficiency measures on reducing the environmental footprint of tourism [85,86]. Similarly, research by Jurkus et al. [87] highlights the crucial role of biodiversity conservation in sustainable tourism practices, aligning with our emphasis on STM2.

**Table 11:** Performance rankings of strategies for green tourism marketing in Guilin.

Strategies	$d_{i}^{+}$	$d_{i}^{-}$	$CC_{i}$	Rank
Eco-friendly infrastructure development (STM1)	11.081	0.759	0.064	2
Biodiversity conservation initiatives (STM2)	9.676	0.567	0.055	4
Waste reduction and recycling programs (STM3)	11.912	0.650	0.052	6
Energy efficiency measures (STM4)	9.567	0.580	0.057	3
Community empowerment and involvement (STM5)	10.316	0.561	0.051	7
Cultural heritage preservation programs (STM6)	10.556	0.498	0.045	9
Social impact and inclusivity campaigns (STM7)	10.644	0.548	0.049	8
Transparent communication and education (STM8)	12.012	0.671	0.053	5
Strategic collaborations and partnerships (STM9)	11.234	0.789	0.065	1

These strategies are essential for reducing the environmental impact of tourism activities and preserving the region’s rich biodiversity. Transparent communication and education (STM8) is ranked fifth, highlighting the importance of effective communication and educational initiatives in promoting sustainable tourism practices. The lower-ranked strategies, including waste reduction and recycling programs (STM3), community empowerment and involvement (STM5), social impact and inclusivity campaigns (STM7), and cultural heritage preservation programs (STM6), may require further attention or enhancements to improve their effectiveness in green tourism marketing in Guilin.

## 5. Discussion, theoretical, and practical implications

The findings of the study highlight significant insights into destination branding for green tourism marketing in Guilin, China. The research utilized the fuzzy AHP and fuzzy TOPSIS methods for analysis. Therefore, the findings of fuzzy AHP analysis indicates communication and brand transparency (GM3) as the most critical aspect, emphasizing transparent communication channels. Following closely is environmental sustainability (GM1), highlighting eco-friendly practices and the preservation of Guilin’s natural environment. Stakeholder collaboration and partnerships (GM4) emerge as the third key criterion. Furthermore, the study explored several strategies using fuzzy TOPSIS method, aimed at influencing consumer perception for green tourism marketing. Firstly, the emphasis on strategic collaborations and partnerships (STM9) echoes the findings of several studies highlighting the role of partnerships between destination management organizations, local businesses, and environmental agencies in promoting sustainable tourism [64]. Secondly, the significance of eco-friendly infrastructure development (STM1) is consistent with existing literature emphasizing the role of sustainable infrastructure in attracting environmentally conscious tourists [88]. Investments in green amenities, energy-efficient transportation, and eco-friendly accommodations not only enhance the visitor experience but also reinforce the destination’s image as a sustainable tourism destination. Furthermore, the emphasis on energy efficiency measures (STM4) and biodiversity conservation initiatives (STM2) reflects the broader trend of destinations prioritizing environmental sustainability [5,70]. These strategies are essential for mitigating the environmental impact of tourism activities and preserving the natural and cultural assets that attract tourists to Guilin.

Previous studies on destination branding and sustainable tourism have contributed significantly to understanding the complexities and challenges within this field [19,20]. Scholars have employed various methodologies to explore different aspects of destination branding, sustainable tourism practices, and their interplay. These studies have provided valuable insights into the factors influencing destination choice, tourist behavior, and the effectiveness of branding strategies in promoting sustainability [89]. Quantitative approaches have been widely used in previous research to examine tourist perceptions, preferences, and behaviors towards sustainable destinations. Surveys and regression analyses have been particularly common, allowing researchers to gather large datasets and statistically analyze the relationships between variables such as environmental attitudes, destination attributes, and travel motivations [90,91]. These studies have shed light on the factors driving tourists to choose sustainable destinations, as well as the potential barriers they may encounter. Qualitative methods, such as case studies and interviews, have been instrumental in exploring the complexities of destination branding strategies and their impact on sustainability initiatives [92,93]. These studies have highlighted the importance of stakeholder collaboration, community engagement, and cultural authenticity in crafting effective destination brands that resonate with tourists and support sustainable development [22]. This research provides valuable insights for assessing and prioritizing criteria, strategies, and performance indicators in sustainable tourism marketing.

### 5.1. Theoretical implications

The findings of this study provide several theoretical implications for the field of sustainable tourism and destination branding, particularly within the context of Guilin, China. This study contributes to the theoretical understanding of sustainable destination branding by identifying and ranking the main criteria and sub-criteria that influence green tourism marketing. The hierarchical importance assigned to these criteria—such as communication and brand transparency, environmental sustainability, stakeholder collaboration, community engagement, and authenticity and cultural relevance—provides a structured framework that can guide future research and practice in sustainable tourism. This adds to the body of knowledge by confirming the paramount importance of clear and transparent communication channels in building a sustainable tourism brand. The fuzzy decision-making approaches represent a significant theoretical contribution to tourism research. These methodologies allow for the handling of the inherent uncertainties and subjectivities in assessing sustainability criteria and strategies. This methodological contribution enhances the precision and reliability of research findings in the field, offering a replicable framework for future studies aiming to assess and prioritize sustainability criteria in different tourism contexts.

### 5.2. Practical implications

The findings of this study offer several practical implications for destination marketers, tourism authorities, and local stakeholders in Guilin, China. The identification of key criteria such as communication and brand transparency, environmental sustainability, and stakeholder collaboration provides actionable insights for tourism marketers. Focusing on transparent communication about sustainable practices will enhance the credibility of a destination’s brand, attracting environmentally-conscious tourists. The research emphasizes the importance of strategic collaborations and partnerships between destination management organizations, local businesses, and environmental agencies. For practical implementation, local authorities and tourism bodies should actively engage in building alliances with businesses that support eco-friendly initiatives, such as hotels with green certifications or transportation services that prioritize sustainability. The study highlights the critical role of eco-friendly infrastructure development in attracting green tourists. Likewise, emphasizing energy efficiency measures and biodiversity conservation aligns with the growing global focus on reducing the environmental impact of tourism. These efforts can differentiate the destination as one that values both tourism and environmental preservation. Finally, tourism stakeholders should view sustainable tourism not just as a marketing tool, but as an integral part of long-term development plans. This research calls for the adoption of sustainability indicators and performance metrics to continuously evaluate the effectiveness of green tourism strategies.

## 6. Conclusion and managerial implications

This study utilized the fuzzy AHP and fuzzy TOPSIS approaches to shed light on the dynamics of sustainable tourism marketing in Guilin, China. The research evaluated the five main criteria, twenty sub-criteria, and nine strategies for molding the destination brand into a beacon of sustainable tourism in Guilin. The prominence of communication and brand transparency is the most critical criterion. This suggests that ensuring openness and clarity in sustainability messaging can significantly enhance the destination’s reputation and attractiveness to environmentally conscious tourists. While environmental sustainability highlights the importance of integrating eco-friendly practices and prioritizing the conservation of Guilin’s natural resources. The findings of fuzzy TOPSIS underline the importance of fostering strategic collaborations and partnerships among stakeholders, spearheading eco-friendly infrastructure development, implementing energy efficiency measures, and championing biodiversity conservation initiatives. These pillars collectively fortify Guilin’s reputation as an eco-conscious destination, poised to attract discerning travelers seeking authentic, environmentally responsible experiences. Additionally, several strategies may rank lower, they nevertheless remain indispensable components of Guilin’s sustainable tourism agenda, warranting continued investment and attention.

### 6.1. Managerial implications

Finally, based on the identified criteria and strategies, the following several managerial implications emerge:

☐ Foster collaborations with local businesses, environmental agencies, government entities, and international sustainability initiatives.
☐ Prioritize investments in eco-friendly infrastructure, including sustainable buildings, energy-efficient transportation, and green amenities.
☐ Encourage the adoption of energy-efficient practices across tourism facilities, accommodations, and transportation services.
☐ Implement targeted conservation efforts and sustainable land use planning to preserve Guilin’s rich biodiversity.
☐ Establish clear and transparent communication channels to convey sustainability efforts and educate tourists about Guilin’s commitment to green tourism.
☐ Launch campaigns and initiatives with positive social impacts, such as community development projects and support for local businesses.

### 6.2. Limitations and future research

This study provides significant understandings for the sustainable tourism development in Guilin. Although, there are several limitations warrant in the study. First, data reliance on subjective judgments in the fuzzy AHP and fuzzy TOPSIS methods may introduce bias, as stakeholder perceptions can vary widely. Future studies could enhance robustness by incorporating more extensive datasets, possibly blending quantitative data from tourist satisfaction surveys, environmental impact assessments, and economic performance indicators. Second, the geographic focus on Guilin may limit the generalizability of the findings to other destinations with different environmental, cultural, and socioeconomic contexts. Future research could apply this model to other regions, allowing for cross-destination comparisons to see if similar priorities emerge or if different strategies prove more effective elsewhere. Additionally, rapid shifts in sustainable tourism trends and technology, such as digital marketing advancements and new sustainable infrastructure innovations, suggest that a longitudinal study could provide insights into how priorities evolve over time. Continuous tracking and updating of criteria and sub-criteria would allow destination managers to adapt to changing tourist expectations and environmental challenges effectively.
